# *Yarrowia lipolytica* and Its Multiple Applications in the Biotechnological Industry

**DOI:** 10.1155/2014/476207

**Published:** 2014-03-13

**Authors:** F. A. G. Gonçalves, G. Colen, J. A. Takahashi

**Affiliations:** ^1^Faculdade de Farmácia, Universidade Federal de Minas Gerais, 31270-901 Belo Horizonte, MG, Brazil; ^2^Departamento de Química, Instituto de Ciências Exatas, Universidade Federal de Minas Gerais, 31270-901 Belo Horizonte, MG, Brazil

## Abstract

*Yarrowia lipolytica* is a nonpathogenic dimorphic aerobic yeast that stands out due to its ability to grow in hydrophobic environments. This property allowed this yeast to develop an ability to metabolize triglycerides and fatty acids as carbon sources. This feature enables using this species in the bioremediation of environments contaminated with oil spill. In addition, *Y. lipolytica* has been calling the interest of researchers due to its huge biotechnological potential, associated with the production of several types of metabolites, such as bio-surfactants, **γ**-decalactone, citric acid, and intracellular lipids and lipase. The production of a metabolite rather than another is influenced by the growing conditions to which *Y. lipolytica* is subjected. The choice of carbon and nitrogen sources to be used, as well as their concentrations in the growth medium, and the careful determination of fermentation parameters, pH, temperature, and agitation (oxygenation), are essential for efficient metabolites production. This review discusses the biotechnological potential of *Y. lipolytica* and the best growing conditions for production of some metabolites of biotechnological interest.

## 1. Introduction

The use of microorganisms to obtain different types of food, such as beer, wine, bread, cheeses, and fermented milk is very old. There have been reports of application of fermentation processes for the production of foods from times before Christ. In the 20th century, the industrial microbiology expanded even more, because they perceived new possibilities for obtaining large variety and quantity of products by fermentative processes. At this time, there was also a boom in the industrial scale production of solvents, antibiotics, enzymes, vitamins, amino acids, and polymers, among many other compounds formed by microbial action [1, 2]. The development of molecular biology techniques in the 1970's gave new impulse in this area, with significant innovations, which have resulted in the emergence of new useful industrial biotechnological processes.

*Yarrowia lipolytica* is an excellent example of a microorganism with multiple biotechnological applications. This nonconventional, aerobic, dimorphic yeast can usually be found in environments containing hydrophobic substrates, rich in alkanes and fats. It can be isolated from cheeses, yoghurts, kefir, soy sauce, meat and shrimp salads. Its maximum temperature of growth is lower than 34°C [3]. It is used in biotechnological and industrial processes for obtaining various products such as citric and isocitric acids and enzymes (acid or alkaline proteases, lipases, and RNase), for bioremediation and production of biosurfactants [4–6].* Y. lipolytica* is also capable of producing *γ*-decalactone, a compound that features a fruity aroma of great industrial interest, obtained by conversion of methyl ricinoleate [7]. Among the metabolites produced by* Y. lipolytica*, one of the most important is lipase, an enzyme that has gained interest of scientists due to its broad technological applications in food, pharmaceutical and detergent production areas. However, the largest industrial application of this yeast species seems to be in the production of biomass to be used as single cell protein (SCP) [3, 8, 9]. Currently there is a great interest in the ability of* Y. lipolytica* to produce and store lipids, which can be used in the production of biofuels, or in the production of oils enriched with essential fatty acids, which has wide application in pharmaceutical and food industries [10].

This multiplicity of applications has resulted in many studies related to lipase production by both wild and naturally occurring strains of* Y. lipolytica* in the presence of lipids [11]. These characteristics enable* Y. lipolytica* to stand among the microorganisms promising for the production of biodiesel. Use of microbial oil in replacement of vegetable oil presents several advantages such as easiness of reaching increased scale, requiring a much smaller area for production, as well as independence of seasonality [12].

*Y. lipolytica* has been considered as a suitable model for studies on the yeasts dimorphism since it produces pseudohyphae filaments in nitrogen-limited conditions [13]. Its growth in fatty acids, as carbon sources, with casein, yeast, or meat extracts induces the formation of hyphae, which is inhibited by the deficiency of magnesium sulfate and ferric chloride, by the presence of cysteine or reduced glutathione levels [3].

The ability of this yeast to grow in alkanes and to hydrolyze triglycerides and fatty acids used as extracellular carbon sources makes it an interesting oleaginous yeast model for studying the metabolism of fatty acids [14].* Y. lipolytica* has developed mechanisms for efficient use of these substrates as carbon sources [15, 16].* Y. lipolytica* produces no ethanol but uses it as a source of carbon, in concentrations up to 3%. In higher concentrations, ethanol becomes toxic to the yeast [8]. It has great potential for production of various intracellular metabolites of industrial importance that are exported by the cells [16].

The growth of* Y. lipolytica* and the secretion of metabolites are affected by different organic ingredients and minerals (and their relative amounts) employed as sources of carbon, nitrogen, and micronutrients and by the pH of the growth medium, incubation temperature, inoculum and intensity of oxygenation [13].

The amount of oxygen available for microbial cells can be provided in the growth medium with the use of compressed air in bioreactors [17], by aeration and agitation in jar flasks [18] and by the addition of oxygen vectors, or by adding a nonaqueous, organic phase to induce a significant increase in oxygen transfer rate [19].

## 2. *Yarrowia lipolytica* and Biotechnological Applications

### 2.1. Bioremediation and Production of Biosurfactants

Bioremediation is a technique that uses microorganisms to speed up the degradation of environmental contaminants into less toxic forms or to promote contaminants reduction to acceptable concentration levels [20–22]. This technique became the principal method used for the restoration of environments contaminated with oil and waste water treatments from the oil industry [15, 23].

Pollution by oil/oil spill is one of the leading causes of environmental damage and can occur in both terrestrial and marine environments or in freshwater [24]. Because of its ability to use alkanes, fatty acids, and oils,* Y. lipolytica* is regarded as a potential agent in bioremediation of environments for the degradation of vegetal and mineral oil waste [15, 24].

There are reports in the literature on the evaluation of indigenous yeasts associated with natural detoxification processes of a wide variety of pollutants. Strains of* Y. lipolytica* have been isolated from several polluted environments. The indigenous microbial populations present in such locations are constantly threatened by the presence of pollutants and, therefore, they have evolved so that their enzyme configuration conquered effectiveness for detoxification [24].

**Figure 1:**
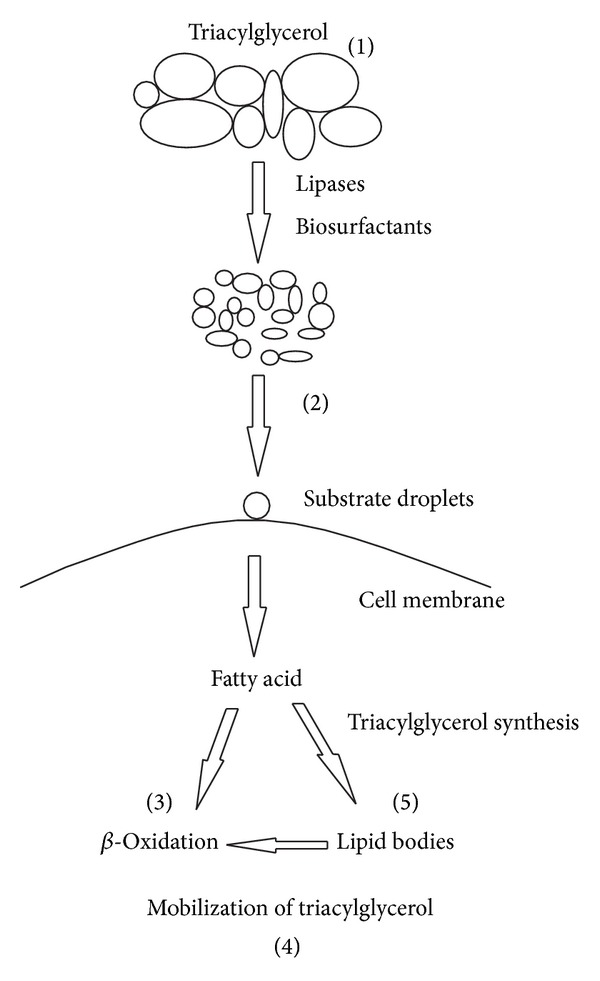
Representation of triglyceride and fatty acid (examples of hydrophobic substrates) assimilation by* Y. lipolytica*. (1) Reduction of droplet size of the hydrophobic substrates by the action of bio-surfactants and extracellular lipase that hydrolyses the triglycerides. (2) Droplets of hydrophobic substrate bound to cell surface protrusions. (3) Fatty acid degradation by *β*-oxidation or (5) triacylglycerol storage lipid bodies. (4) Mobilization of triacylglycerol by lipases (adapted from Beopoulos et al. [25]).

Under the evolutionary point of view, it is believed that microorganisms that multiply in aqueous environments rich in materials of hydrophobic nature, dispersed in the environment in the form of drops, developed a mechanism to facilitate the use of such substrates as carbon sources. The ability of* Y. lipolytica* in degrading a variety of organic compounds, including aliphatic and aromatic hydrocarbons, is always accompanied by the production of bio-surfactants, molecules made up predominantly of glycolipids, which increases the contact surface. The growth of microorganisms in hydrophobic substrates requires a contact between the hydrophobic substrate present in the organic phase and the cell surface. This contact may occur by direct adsorption of hydrophobic droplets on the surface of the cell or by the action of bio-surfactants and both mechanisms are reported for* Y. lipolytica*. The interaction between the hydrophobic molecules and cells is mediated by proteins or glycoproteins present in the cell wall and the surfactant that is secreted can facilitate this interaction [13, 15]. According to Beopoulos et al. [25], the extracellular lipase produced by* Y. lipolytica* also acts on the hydrophobic substrate, permitting the hydrolysis of triglycerides. The action of bio-surfactant and lipase occurs progressively, after formation of several droplets that facilitate the transport of the substrate (Figure 1).

The bio-surfactants are molecules that possess both hydrophilic and hydrophobic groups, which determine properties such as adsorption, formation of micelles, macro- and micro emulsions, and detergency solubilization capacity, indispensable for the process of bioremediation [26].

The composition and characteristics of bio-surfactants produced by microorganisms are influenced by the nature of the carbon and nitrogen sources used, by the presence of phosphorus, iron, manganese, and magnesium in the means of production, temperature, pH, and agitation. The production can be spontaneous or induced by the presence of lipophilic compounds, by changes in pH, temperature, aeration and agitation speed and subjection to stress conditions (e.g., low concentration of nitrogen) [27].

Cirigliano and Carman [28] studied different carbon sources (hexadecane, paraffin, soy oil, olive oil, corn oil, and cottonseed oil) for the production of bio-surfactants by* Y. lipolytica* and realized that there was greater productivity when hexadecane was used. Sarubbo et al. [29] studied the production of bio-surfactants by* Y. lipolytica* from glucose and reported that the presence of hydrocarbons was not a prerequisite for biosynthetic induction of surfactants under the conditions used.

### 2.2. *γ*-Decalactone

**Figure 2:**
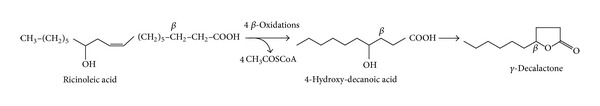
Ricinoleic acid bioconversion to *γ*-decalactone (adapted from Schrader et al. [33]).

The *γ*-decalactone is a peach-scented compound widely used in food and beverages, which is the reason of the great interest in its biotechnological production [13]. This can be accomplished by biotransformation of ricinoleic acid by* Y. lipolytica* [30]. Ricinoleic acid is a hydroxylated fatty acid (C18) and, in its esterified form, is the main constituent of castor oil. This fatty acid is the precursor of *γ*-decalactone [13]. To increase the availability of ricinoleic acid to cells, castor oil can be hydrolyzed by lipases [30], generating esters such as methyl ricinoleate [13]. The process involves the degradation of ricinoleic acid into 4-hydroxy-decanoic acid, a precursor of *γ*-decalactone, which is obtained by the action of peroxisomal *β*-oxidation enzymes (Figure 2) [31]. Peroxisomal *β*-oxidation occurs in four cyclic oxidation reactions catalyzed by the enzymes acyl-CoA oxidase and 3-ketoacyl-CoA thyilase [31, 32].

*Y. lipolytica* produces high yield of *γ*-decalactone and also has a large number of genes encoding enzymes that degrade hydrophobic substrates and, therefore, became a model for this metabolic route.* Y. lipolytica* has a family of six medium-chain acyl-CoA oxidases, the enzyme that catalyzes the first reaction of *β*-oxidation in this series [31].

### 2.3. Production of Citric Acid

Citric acid is much employed in industry as acidulant to stiff aromas in food, beverages, and pharmaceuticals as well as preservative in cosmetics. To feed the industrial demand, citric acid has been produced by fermentation processes since the beginning of the 20th century [34]. Its annual production reached approximately 1.6 million tons in 2008 [35], being one of the metabolites most produced in industrial scale [36].

The most commonly used microorganism for citric acid production is* Aspergillus niger* [34]. Species of yeasts as* Y. lipolytica*,* C. guilliermondii* and* C. oleophila* have also been used [37],* Y. lipolytica* being the most used yeast [38].

The microorganism, the type of substrate, and growing conditions (temperature, aeration, concentration and source of carbon and nitrogen, phosphate, trace elements, and pH) are factors that influence directly the production of citric acid [39–41]. In general, this production is conducted by batch, fed batch or continuous process [37]. The water content of the culture medium or, in other words, the type of fermentation, solid substrate or submerged fermentation, also influences the production of citric acid. Therefore, to obtain optimum performance, it is mandatory to evaluate the performance of the strain in different conditions [42].

According to Papanikolaou and Aggelis [36], the production of citric acid mainly occurs when the concentration of nitrogen in the culture medium becomes limited and using sugars or glycerol as carbon sources. Nitrogen limitation causes rapid decrease in the concentration of AMP (adenosine monophosphate), responsible for the formation of intracellular enzyme NAD^+^ (nicotinamide adenine dinucleotide) isocitrate dehydrogenase (that turns isocitric acid in alpha-ketoglutaric acid), causing loss of enzyme activity. As a consequence, in the build-up of citric acid in the mitochondria of the cell, upon reaching a critical level, it is secreted in the cytosol.

#### 2.3.1. Influence of Carbon Source on Production of Citric Acid

Carbon is the chemical element present in higher amounts in the cell. The accumulation of citric acid is strongly influenced by both the carbon source and its concentration. Yeasts are able to produce citric acid using a variety of carbon sources of various types [43].

Many species of yeasts that grow in carbohydrate substrates are capable of producing high concentrations of citric acid [43].* Y. lipolytica* is the unique yeast able to maximize the production of citric acid using both carbohydrate and fat carbon sources [37]. Many carbon sources have been screened for production of citric acid by* Y. lipolytica* such as glucose [40], n-paraffin [44], ethanol [38, 45], glycerol [37, 46, 47], sunflower oil [48], and wastewater from the production of olive oil [49].

Glucose and yeast extract are considered preferred sources of various microorganisms, which is not surprising since yeast extract contains all the necessary micronutrients, including metal ions, for microbial growth [50]. The presence of carbohydrates easily absorbed by microorganisms is considered to be a key point for citric acid production in significant quantities. Among these carbohydrates, sucrose is considered the best source of carbon, followed by glucose, fructose and galactose [42].

Some authors support the use of cheaper substrates, such as agroindustrial wastes, to produce citric acid [36, 42, 44]. Glycerol is also an interesting substrate to be used in the production of citric acid since in the process of esterification, for each 10 kg of biodiesel produced, 1 kg of glycerol is formed as a by-product [36, 47]. Molasses, a by-product of the sugar and alcohol industry, containing high content of sugars (around 40–55%) can also be used for the production of citric acid [42]. The use of wastewater from the processing of olives, enriched with glucose, was proposed by Sarris et al. [49], as a substrate for producing citric acid. Crolla and Kennedy [44] studied the effect of n-paraffin in the production of citric acid by* C. lipolytica* NRRL-Y-1095 and obtained significant amounts of citric acid and biomass. The use of n-alkanes usually provides great citric acid performance. In addition, paraffinic substrates are very suitable sources for obtaining biomass (single-cell proteins). To obtain high concentration of citric acid, the concentration of the carbon source is also important [34, 40]. This is corroborated by Sarris et al. [49]; evaluating different strains of* Y. lipolytica* for citric acid production in carbon limiting conditions, this group noted that although there is formation of biomass, only low yield of citric acid was obtained due to the insufficient amount of carbon present in the growth medium. Finogenova et al. [45] tested the effect of ethanol on the production of citric acid by a mutant strain of* Y. lipolytica* N1, getting maximum output when using ethanol concentrations in the range between 0.01–1.0 g/L.

On a growth medium containing 200 g/L of crude glycerol, the mutant strain of* Y. lipolytica* Wratislavia AWG7 produced an exceeding of 0.69 g of citric acid/g glycerol consumed. A lower yield was obtained by* Y. lipolytica* Wratislavia K1 (about 0.45 g/g) that also produced erythritol in high yield, thus reducing the production of citric acid [51].

#### 2.3.2. Influence of Nitrogen Source on Production of Citric Acid

The nitrogen source (type and concentration) also influences the production of citric acid by microorganisms. Physiologically, there is a preference for salts, such as ammonium nitrate, urea and ammonium sulfate, peptones, and malt extract, among others. Acidic compounds of ammonia are also well accepted, since its consumption causes decrease in pH of the medium, which is essential for citric fermentation. However, in the early hours of fermentation, the ideal is that the pH value does not change much to ensure biomass formation [42].

It is consensus in the literature that the production of citric acid by yeasts is favored under nitrogen-limiting conditions, a situation that leads the organism to citric acid production and lipids [34, 36, 45, 49, 52]. Production of citric acid requires nitrogen concentration in the range of 0.1 to 0.4 g/L. Higher concentrations promote cell growth, but decrease the production of citric acid [42].

Anastassiadis et al. [34] identified the ammoniac nitrogen as limiting substrate for citrate production. According to the results obtained, the production of citric acid begins a few hours after nitrogen exhaustion. The authors even contradict many reports in the literature, reporting that is not the extracellular nitrogen exhaustion that takes the production of citric acid but the limitation of intracellular nitrogen, accompanied by increasing intracellular levels of ammonium ion and energy (ATP). These conditions would induce a specific active transport system for the secretion of citrate. According to the authors, this event may be explained by analyzing* Candida oleophila* biomass. During the phase of secondary metabolites production (idiophase) which corresponds, in general, to the stationary phase of microbial growth, the yeast cells increase in size. This would occur also with their vacuoles, organelles that accumulate citric acid, which are secreted when the active citrate transport system is induced. This induction happens when there is limitation of intracellular nitrogen, followed by high concentrations of intracellular ammonium ion and energy. According to Anastassiadis et al. [34], the increase in the intracellular concentration of ammonium ion in* C. oleophila* can be explained by proteolysis that occurs as a result of intracellular nitrogen limitation, which would lead to the extracellular exhaustion. This limitation would cause conversion of glucose in citrate, in order to obtain energy.

#### 2.3.3. Effect of Temperature, pH and Oxygenation on the Production of Citric Acid

The fermentation temperature is a variable that affects directly in the production of citric acid. To determine the optimum temperature, it is usual to accept a variation of only ±1°C, to ensure an effective process [44]. The pH of the growth medium has an important influence on the production of citric acid since it affects the metabolism of* Y. lipolytica*. Changes in pH, along with the time of cultivation, are influenced mainly by the microorganism used [42], by the technique employed and by the nature of the substances produced. In the production of organic acids, such as citric acid, there is a decrease in the pH of the medium [41, 42].

Karasu-Yalcin et al. [41] achieved high yield of citric acid from strains of* Y. lipolytica* at an optimum temperature of 30°C, while Crolla and Kennedy [44] reported that the optimum temperature for both citric acid production and biomass formation by* C. lipolytica* was 26–30°C. In Karasu-Yalcin et al. [41] studies, the* Y. lipolytica* (NBRC 1658 and a domestic strain 57) strains showed optimum production of citric acid at initial pH values of 7.0 and 5.2, respectively. On the other hand, Kamzolova et al. [48] reported that the pH of the medium influenced both the production of citric acid and isocitric acid.* Y. lipolytica* produced similar amounts of these acids at pH value of 4.5. However, at higher pH (6.0), the yeast produced higher amounts of isocitric acid. This occurred because the citric acid transport across the membrane is stimulated at low pH values, while the isocitric acid transport is independent of the pH of the medium [53].

As is known, oxygen is essential for aerobic bioprocess. Microbial growth in a reactor depends on the oxygen transfer rate, which is widely used to study the behavior of microorganisms. An increase in the availability of dissolved oxygen in the culture often results in improving the yield of secondary metabolites [19]. Kamzolova et al. [5] reported that need of oxygen by* Y. lipolytica* N1 for growth and for citric acid production, depended on the iron concentration in medium. The effect of iron and oxygen concentrations affected the functioning of the electron transport chain in the mitochondria of yeast. According to the authors, when it was applied a relatively low pressure (20%) and furnished a high concentration of iron (3.5 mg/L), yield of citric acid production increased (120 g/L).

#### 2.3.4. Inductors and Inhibitors of Citric Acid Production

Attention should be given also to trace elements, which should be strictly controlled. Bivalent metal ions, such as zinc, manganese, iron, copper, and magnesium, affect the production of citric acid [42]. Iron salts are also essential, because they activate the production of acetyl coenzyme A, precursor of citric acid. However, excess iron activates the production of aconitase, enzyme that catalyzes the isomerization of citrate to isocitrate, directing the reactions to formation of isocitric acid, therefore reducing production of citric acid [44]. Finogenova et al. [45] described that, for the production of citric and isocitric acids by mutant strain of* Y. lipolytica* N1 grown in ethanol, high concentrations of zinc and iron were necessary. On cultivation conditions where zinc concentration was limited, cell growth was low and there was no production of citric acid. When zinc was added in the medium using nitrogen-limited conditions, production of citric and isocitric acids increased considerably. Similar behavior was observed in relation to the effect of iron concentration on the production of citric acid. Under nitrogen-limiting conditions, an increase in the intracellular iron content from 0.13 to 2.5 mg/g resulted in increased production of citric acid. But, with the increase in iron concentration from 0.25 to 0.48%, there was observed a decrease in the production of citric acid. Soccol et al. [42] also claim that low levels of phosphate in the medium have a positive effect on yield of citric acid since phosphate would act on the enzymatic regulation of acid production.

### 2.4. Accumulation of Lipids

Oleaginous microorganisms have the ability to transform organic acids into acetyl-CoA, an intermediate that is used for the biosynthesis of lipids [54]. The lipid accumulation primarily depends on the physiology of the microorganism, nutrient limitation and environmental conditions such as temperature and pH. These factors also affect the production of other secondary metabolites, such as ethanol and citrate [12, 50, 55]. Beyond* Y. lipolytica*, the mainly oleaginous microorganisms are of the genera* Candida, Cryptococcus*,* Rhizopus*, and* Trichosporon*, and lipid profile produced differences between the species [25, 56]. On average, these yeasts accumulate lipids in a quantity corresponding to 40% of their biomass. In conditions in which there is limitation of nutrients, the accumulation of lipids can reach values that exceed 70% of their biomass.

*Y. lipolytica* is one of most interesting oil-producing yeasts. Wild or genetically modified strains of yeast species have been reported as being capable of producing large amounts of intracellular lipids, which are stocked in the lipid bodies, during t growth in various types of hydrophobic materials [16]. In spite of accumulating fewer lipids than some other oleaginous yeast species,* Y. lipolytica* is the only one able to accumulate large amounts of linoleic acid, representing more than 50% of the fatty acids accumulated by the yeast [12].

#### 2.4.1. Lipid Synthesis

The intracellular oil accumulation is a consequence of yeast metabolism imbalance. When all the nutrients are present in suitable amounts in the culture medium, there is new cell synthesis, that is, microbial growth. But when the microorganism is deliberately deprived from any essential nutrient, lipogenesis induction occurs, namely, production and storage of oil [55, 57].

Lipids can be stored inside the cell by two different routes: (1)* de novo* synthesis, which involves the production of fatty acid precursors, such as acetyl and malonyl-CoA and their integration in lipid storage biosynthesis and (2) via accumulation ex novo, which involves the capture of fatty acids, oils and triglycerides of the growth medium, followed by their accumulation within the cell. This requires the hydrolysis of hydrophobic substrates on the outside of the cell, fatty acid transport to the interior and reassimilation as triglycerides and esters, followed by their accumulation in lipid bodies [12].

**Figure 3:**
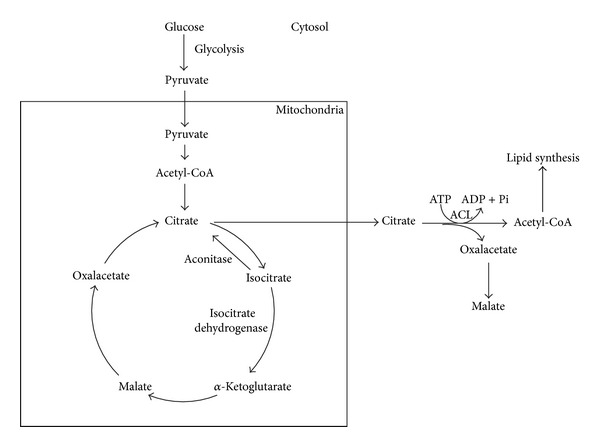
Synthesis of lipids by citrate and excess nitrogen limitation. Scheme of the major metabolic pathways for lipid synthesis in* Y. lipolytica*. The glucose undergoes glycolysis and enters in the mitochondria as pyruvate to be used in the tricarboxylic acid cycle. Excess acetyl-CoA is transported from the mitochondria to the cytoplasm in the form of citrate. The cytosolic acetyl-CoA is the precursor for the synthesis of lipids in the lipid bodies. Adapted from Rossi et al. [55] and Tai and G. Stephanopoulos [10].

When there is nutrient limitation, some metabolic pathways are repressed due to the synthesis of proteins and nucleic acids, while others are induced (synthesis of fatty acids and triglycerides). During the growth phase, nitrogen is essential for the synthesis of proteins and nucleic acids, required for cell proliferation. When nitrogen is limited, this process is slowed and the growth rate declines rapidly. The excess carbon is then piped to the synthesis of lipids, which leads to an accumulation of triglycerides in lipid bodies. During this phase of stocking, precursors (acetyl-CoA, malonyl-CoA, and glycerol) and energy (ATP and NADPH) are necessary for lipids synthesis [12, 50, 55]. This process is known as* de novo* synthesis, which occurs in oleaginous microorganisms. The process of lipids accumulation inside cells of oleaginous microorganisms is also related to production of citric acid, according to Figure 3.

The reactions of the citric acid cycle begin with the condensation reaction by an enzyme which catalyzes the acetyl CoA in the cycle, with the formation of citric acid (or citrate). Then, with the action of the enzyme aconitase, citric acid is acid-isomerized. Soon after, there is a reaction of oxidative decarboxylation of isocitrate that is catalyzed by the enzyme isocitrate dehydrogenase and isocitrate oxidized and decarboxylated to *α*-ketoglutarate. This reaction is reversible; that is, depending on the situation, formation of *α*-ketoglutarate or reductive carboxylation of *α*-ketoglutarate, forming isocitrate. This enzyme, isocitrate dehydrogenase occurs, is affected by adenosine monophosphate (AMP), a positive stimulator of the enzyme, which has the role of regulating dehydrogenase activity. With high concentration of ATP, the activity of isocitrate dehydrogenase decreases. Upon ATP consumption, there is an increase in AMP concentrations, which stimulates the rate of isocitrate oxidation. Citrate and isocitrate concentrations are increased when the activity of the enzyme isocitrate dehydrogenase decreases. This happens because the equilibrium constant of aconitase reaction greatly favors citric acid accumulation [58].

With nitrogen limitation, occurs activation of AMP deaminase that fills the cell lacking of ammonium ion. As a result, the concentration of mitochondrial AMP decreases, causing the fall of activity of citrate dehydrogenase. The citric acid cycle is then blocked at the level of isocitrate, which accumulates and is balanced with the citrate action of aconitase. The citrate goes into the cytosol and is cleaved by ATP citrate lyase (ACL), giving rise to acetyl-CoA and oxaloacetate, which is the precursor of fatty acids biosynthesis. The acetyl-CoA excess is therefore the key element for the* new* synthesis of lipids in oleaginous microorganisms [25, 58].

When nonoleaginous microorganisms are submitted to nutrient limitation, cell growth also tends to end, but the carbon present in the medium is converted into several other polysaccharides, such as glycogen, glucans and mananes [12].

New extra- and intracellular fatty acids, previously absent in the substrate, can be produced by oleaginous yeast fermentation under certain conditions. An important application of new fats with polyunsaturated fatty acids (PUFA) is as food or nutritional supplements. However, the most important application of these metabolites consists in the production of lipids with high added value, such as in the production of exotic high-value fats such as Shea butter [59].

Lipids produced by* Y. lipolytica* represent an attractive source of edible oils [60] and have been considered as an alternative source for the production of PUFA, cocoa butter substitutes (CBS), and structured lipids. Papanikolaou et al. [61] studied the production of lipids by* Y. lipolytica* detecting stearic, oleic, linoleic, and palmitic acids. In all cases, the microorganism demonstrated ability to increase the concentration of stearic acid, even if this fatty acid was not present in high concentrations in the substrate. This ability allowed the synthesis of an interesting profile of lipids with high percentages of stearic, palmitic and oleic acids, and composition similar to cocoa butter.

The microorganisms begin to accumulate lipids when there are restrictions, principally of nitrogen, in the form of ammonium ion in culture medium and excess carbon source such as glucose [12, 50, 55, 62]. The fatty acids profiles produced, the amount, the productivity, and the efficiency of the conversion are also influenced by several factors during the fermentation process, such as the type of substrate used, choice of limiting nutrient, temperature, pH and aeration [50, 61, 63, 64]. The carbon source is the main factor that influences the lipid composition of the oils produced by yeasts [63, 65].

Athenstaedt et al. [64] cultivated* Y. lipolytica* in a medium whose unique carbon source was glucose and replaced it by oleic acid. This resulted in the change of lipid composition, as well as increased lipid production.

Currently, the cost of microbial oil production is greater than that of plant and animal oils. However, there are features to lower the cost of the production process of this type of oil. Using alternative carbon sources and as a result, increasing accessibility, is an alternative [54], since this factor corresponds to 80% of the total cost of biodiesel produced from microbial lipids, when using glucose as carbon source [57, 66].

The use of fermentable carbohydrates such as glucose is needed, but these carbohydrates can be obtained also by the hydrolysis of cornstarch or similar raw materials. Another alternative of carbon sources is the molasses of sugar cane and beet sugar, rich in sucrose, but not all oil-producing yeasts can metabolize them. The oleaginous yeasts are known for using these two types of sugars simultaneously [57]. When glycerol is added to dextrose or xylose in culture medium, greater amounts of unsaturated fatty acids are produced while glycerol activates the expression of different enzymes used in the synthesis of these acids [65].

The most commonly used nitrogen sources are yeast extract, peptone, nitrate, and ammonium sulfate [16, 60, 61, 67] but combination of nitrogen sources is also commonly used [11, 49]. The critical nitrogen concentration in the medium to induce lipid accumulation in* Y. lipolytica* is 10^−3^ mol/L [68]. It is important that nitrogen concentration not exceed this value, to avoid production of other metabolites (citric acid), therefore affecting production and accumulation of lipids [12, 69].

In addition to nitrogen, other nutrients may be limited to induce the production of lipids, such as metal ions magnesium, iron, zinc or phosphorous [12, 50].

The pH and temperature are parameters that must be controlled in the growth medium for lipid production, once that interfere in the production and storage of oil inside cells. In the literature, the most pH values used for the cultivation of* Y. lipolytica* with purpose to produce oil are included in the range between 5.0 and 7.0 [11, 49, 54, 60, 61]. Papanikolaou et al. [60] observed mainly cell growth using initial pH 6.0. In relation to the temperature, it is reported that the optimum temperature for lipase production by* Y. lipolytica* is in the range between 28 and 30°C [11, 49, 54, 60, 61]. As these yeasts are aerobic, they need to be grown in bioreactors with agitation and aeration, usually between 100 and 250 m^3^ [57].

#### 2.4.2. Carbon and Nitrogen Ratio in the Growth Medium for Accumulation of Lipids

The excess carbon and nitrogen deprivation are essential factors for the induction of lipogenesis in oleaginous microorganisms. The use of media with the proper C/N ratio is essential to maximize the production of lipids. In a batch process, converting lipid carbons will depend on the duration of the growth phase that depends on the C/N ratio in the fermentation medium [55].

In some oleaginous fungus, excess of carbon source in the medium leads to over production of organic acids, such as pyruvic acid and several other products, to the detriment of lipids accumulation [55]. In the case of* Y. lipolytica*, in the conversion of glucose into lipids, at high initial C/N ratio (80–120 C mol/mol N), cell growth can be followed by citric acid production, leading to low lipid accumulation [12].

Fontanille et al. [54] cultivated* Y. lipolytica* on glucose and glycerol, keeping the ratio C/N equal 62 (80 g/L of C and 3 g/L of N). After 48 h of fermentation, they obtained about 16 g/L of lipids, achieving a 20% conversion yield of carbon into lipids. Najjar et al. [11] also cultivated* Y. lipolytica*, using two different culture media, the first one containing glucose and olive oil (YPDO) and the other containing only olive oil (YPO). In both media, the nitrogen sources used were bacteriological peptone and yeast extract. In the medium YPDO, glucose was the main source of carbon and energy responsible for cell growth. Using YPO, the free fatty acids and triglycerides stocked were consumed and used to increase the concentration of biomass.

The C/N ratio used by Easterling et al. [65] was 10 : 1 for the production and accumulation of lipids by yeast* Rhodotorula glutinis*. The authors aimed at studying the influence of different carbon sources (dextrose, fructose and glycerol, and xylose) on lipid production by* R. glutinis* and observed that the amount of accumulated fat was increased by using glycerol more than using dextrose and glucose. With glycerol as a carbon source, the lipid production increased by of average 13%, whereas using dextrose or xylose as a substrate, the lipid accumulation decreased by about 9% for both substrates. The efficiency of the conversion of glucose into fat increased from 0.25 to 0.40 (mol/mol glucose lipids), when the C/N ratio was increased from 150 to 350 (mol/mol glucose nitrogen) for the yeast* R. glutinis*. However, when the ratio C/N exceeded 350 g C/g N, due to severe nitrogen deficiency, there was a rapid decrease in cell viability before they reached lipid accumulation stage [12].

### 2.5. Lipase Production

Lipases are among the most interesting primary metabolites produced by* Yarrowia*, mainly because of the various possibilities for industrial applications. Lipases of yeasts do not present region selectivity in relation to the position of fatty acids on glycerol molecule and have potential application in the synthesis of esters of short chains, which can be used as flavoring compounds [13, 70–73].* Y. lipolytica* is able to produce both intracellular and extra lipases [74, 75]. Several factors influence the synthesis of lipase by microorganisms. The main factors are carbon and nitrogen sources, presence of inductors, stimulators, or inhibitors, agents affecting the oil/water interface, incubation temperature, and pH of the culture medium and inoculum [71, 76–78].

Lipase production reaches a peak when the culture enters the stationary phase of growth [73]. Lipase activity declines rapidly after reaching the maximum level, because of proteolysis, catalyzed by proteases that are formed during the phase of cell growth and released with the cell lyse [79].

Composition of the culture medium, in particular in the incorporation of different lipid substances, may result in the production of isozymes [79]. Isozymes are enzymes that occur in more than one form, differing in their structures, but that catalyze the same reaction. Enzymes can be inductive loads, that is, produced by microorganisms in the presence of an inductor, which may be the substrate or product of their hydrolysis [80, 81].

#### 2.5.1. The Effect of Carbon Source on Production of Lipase

The carbon source present in the culture medium can stimulate or inhibit the synthesis of lipase. Some fats or oils, as well as carbohydrates, organic acids, glycerol, and other alcohols and fatty acids have been used as inductors of lipase production by* Yarrowia* [71, 82]. The lipase activity is usually detected in growth medium containing lipid materials such as olive oil, soybean oil, tributyrin and oleic acid, which suggests that the production of the enzyme is induced by these substrates [83–85]. On the other hand, unrelated substrates to fats and oils such as carbohydrates, provide good cell growth but are not good for synthesis of lipase [79, 86].

In the study by Obradors et al. [76], oleic acid showed efficient biomass yield and lipase production in relation to other fatty acids with different sizes of carbonic chain. Tan et al. [82] reported that vegetable oils containing oleic and linoleic acid were the best for the biosynthesis of lipase from* Candida* species. Najjar et al. [11] also observed that the olive oil induced the production of lipase by* Y. lipolytica*, when it was used as a sole source of carbon. But when olive oil was added along with the glucose in the culture medium, glucose acted as an inhibitor of lipase production. Low production continued until glucose was fully consumed, being detected late in reaching the peak of enzyme production. The olive oil is widely used as carbon source for production of lipase, since it contains about 70% oleic acid (C18:1). Montesinos et al. [77] reported that when olive oil is used as sole source of carbon, the microorganism follows a sequential mode. Olive oil is initially hydrolyzed by the residual lipase present in the inoculum. Then the microorganism consumes glycerol released as carbon source, but even without producing lipase. Finally, the free fatty acids are consumed, acting as inducers of the formation of a significant amount of lipase.

Yeasts are able to respond to stimulus and are constantly monitoring and adapting themselves to the medium in which they are grown. In the presence of oleic acid, yeast cells induce the expression of a set of enzymes needed for the reactions of *β*-oxidation of fatty acids and proteins that are involved in the expansion of the peroxisomal compartment and participating in the respiratory chain, for energy production [87]. Even under these conditions, there is a direct regulation of the genes that express lipase, by specific genes to oleic acid, identified as SOA genes (specific for oleic acid). These genes encode proteins that control gene expression LIP-2, responsible for the extracellular lipase by* Y. lipolytica* [14].

The literature shows that free fatty acids from triglycerides hydrolysis, mainly oleic acid, are better lipase inducers of the triglycerides and this is valid not only for* Y. lipolytica* but also for other yeasts. Other fatty acids are also reported as inducing the production of lipase [71, 75, 77, 88].

The complex culture media, containing protein hydrolysates such as peptones, yeast and malt extracts are used in fermentation processes but are very expensive. An alternative to decrease the costs would again be the use of agroindustrial wastes, inspite of being required to conduct nutrient supplementation using such culturing materials [89, 90].

#### 2.5.2. Choice of the Nitrogen Source for Production of Lipase

The nitrogen source is a factor that has shown great influence on the lipolytic enzymes. Both the organic and inorganic nitrogen have an important role in the synthesis of the enzyme [82, 89]. Among nitrogen compounds used for lipase production, the most common sources that have been hydrolyzed proteins, peptones, amino acids, yeast and malt extracts, urea, nitrate and ammonia salts, agroindustrial waste, such as corn and water soy flour [71, 77, 89]. The tryptone, obtained by the hydrolysis of casein by trypsin, and yeast extract increases the production of lipase and cell growth. Generally, nitrogen sources are widely used because they provide amino acids and vitamins are enzyme cofactors, which are essential for cellular physiology [91].

The selection of the most suitable nitrogen source depends on the microorganism used and association with other ingredients of the culture medium [77]. To verify the influence on the growth of* Y. lipolytica* lipase production, various sources of nitrogen (organic and mineral) were tested by Shirazi et al. [83]. The sources of mineral nitrogen did not demonstrate significant effect on the growth of yeast in the production of lipase. However, increased production of lipase in media containing certain organic nitrogen sources has not been observed. The largest production of lipase was obtained in the presence of tryptone N1, a casein hydrolysate, which is rich in amino acids and peptide-free, compared with other nitrogen-containing organic or mineral substrates. In this way, the use of tryptone N1 can be considered interesting technological point of view, although its cost is high [75].

Almeida et al. [91] also tested the effect of mineral and organic nitrogen sources in the production of lipase and* Candida viswanathii* growth. They noted that one of the mineral sources, the chloride, and ammonium sulfate provided good cell growth, but had no effect on lipase production. However, the ammonium nitrate influenced positively both the growth and production of lipase. The organic sources (tryptone, peptone and yeast extract) increased lipase activity and cell growth, the yeast extract is considered the best source of nitrogen.

Among all organic nitrogen sources tested, the acid hydrolysate, composed exclusively of free amino acids, but low in tryptophan, yielded low lipase production, while the other enzymatic hydrolysates of casein led to high yield of production. Fickers et al. [75] suggest that specific peptides in enzymatic hydrolysates can regulate the production of the enzyme with lipase activity.

#### 2.5.3. Carbon and Nitrogen Ratio (C/N) and Other Nutrients for Lipase Production

The production of microbial enzymes also depends on the carbon-nitrogen ratio [99]. A balanced culture medium can contain ten times more carbon than nitrogen. This ratio 10 : 1 ensures high protein content, while a higher ratio such as 50 : 1 favors the accumulation of alcohol, metabolites derived from acetate, and extracellular polysaccharides or lipids [100].

In general, carbon, hydrogen, oxygen, nitrogen, sulfur, phosphorus, magnesium and potassium are required in large quantities because they participate in almost all of the cellular substances. Some elements (sulfur, phosphorus, magnesium and potassium) should be provided in the form of salts [101].

The mineral elements (phosphorus, sulfur, potassium, calcium, magnesium, sodium, iron and chlorine) and a small amount of trace elements (manganese, copper, zinc, molybdenum, chromium, nickel, cobalt and boron), which play an important role as constituents of enzymes and coenzymes are usually needed in the culture media for production of enzymes for* Y. lipolytica* [102].

According to Tan et al. [82], the metal ions actually interfere with the synthesis of lipase. According to the authors, magnesium ions, potassium and sodium are beneficial for the biosynthesis of lipase, while the calcium inhibits because form complexes with the fatty acids, changing its solubility and behavior in the oil/water interface.

#### 2.5.4. Water Activity

The amount of water available in the culture media for* Yarrowia* interferes directly in cell growth and productivity. Many studies of lipolytic enzymes production by yeasts have been conducted in submerged crops, that is, in liquid cultures, with high water activity which is identified as submerged fermentation (SmF). The production of microbial enzymes can also be made by solid substrate fermentation process (SSF). A solid-state culture can be defined as the one in which there is microbial growth in solid material, with low amount of free water. The use of solid media allows a fast population growth of filamentous fungi, allowing the detection of specific enzymes [78, 103].

By-products generated in food processing, rich in fatty acids, triglycerides and/or sugars [78] have been used for the production of lipase by yeasts grown on semisolid culture medium. Using these raw materials, the total capital invested in the production of lipase is significantly lower in strong cultures than in submerged ones. However, one of the problems with this method is low oxygenation, which destabilizes the aerobic processes.

#### 2.5.5. Effects of Temperature, pH and Oxygenation in the Production of Lipase

The temperature affects the microbial growth parameters as the adaptation time (lag phase), the specific growth rate and total income, and influences the biosynthesis of primary and secondary metabolites [100]. Corzo and Revah [96] detected that the temperature was the factor that most influenced lipase production by* Y. lipolytica* 681. The optimum temperature for the detection of lipase activity was 29.5°C.

The hydrogen ion concentration in a culture medium can affect microbial growth indirectly, affecting nutrient availability or directly, acting on cellular surfaces [100].

It is commonly reported that lipase production by liquid substrate fermentation, using different microorganisms, is accomplished when lowering pH occurs at the end of cultivation [70, 96, 104, 105].

Lopes et al. [17], using a pressurized bioreactor for* Y. lipolytica* cultivation, found that the increased availability of oxygen caused induction of antioxidant enzyme superoxide dismutase, a defense mechanism of the cells against oxidative stress. The increased pressure has not affected the ability of lipase production, but using air pressure at 5 bar, there has been an increase of 96% of extracellular lipase activity.

In another study also involving* Y. lipolytica*, the authors added perfluorocarbon (PFC) to the growth medium, to increase the availability of oxygen to the yeast and thus increase productivity of lipase. Higher rates of growth of* Y. lipolytica* were found with increasing concentration of PFC and upon increasing agitation speed. Lipase production by yeast was increased 23 times with addition of 20% (v/v) of PFC to 250 rpm. In addition, it was demonstrated that the use of PFC along with glucose is more effective in the production of lipase than the conventional use of olive oil, precisely because of the increase in oxygen transfer [18].  Table 1 shows the types of substrates, nitrogen sources and fermentation more used for* Y. lipolytica* cultivation for production of citric acid, lipids, lipases and biomass.

## 3. Conclusions

There is an expectation around* Y. lipolytica*, which is considered as a promising and efficient microorganism to be used by biotechnological industry. Its major application stands out due to its rich and varied biosynthetic potential, being capable of producing various types of metabolites. Additionally, simple alterations in the fermentative process can be accomplished in order to modulate metabolites production by this species. The versatility of the metabolites produced, along with the easiness of scale increasing, is raising numerous proposals for industrial applications of* Y. lipolytica* in the most diverse segments, mainly in the sectors of energy, foods and pharmaceutical industries that move a great volume of the world economy. To make the production of these metabolites industrially viable, it is important to obtain detailed knowledge of suitable growing conditions of* Y. lipolytica*. Investments in the optimization of the metabolic production, as well as in the operationalization of the fermentative processes and the improvement of the producing strains, by mutation or by selecting new strains, should soon result in novel marketed products from this microorganism.

## Figures and Tables

**Table 1:** Substrates, nitrogen sources, type of fermentation used to produce citric acid (CA), lipids, lipase, and biomass by *Y.  lipolytica*.

Substrate	Nitrogen source	Fermentation	Product	Reference
Glucose	Ammonium nitrate and yeast extract	SmF	CA	Antonucci et al. (2001) [40]
Ethanol	Ammonium sulphate and hydrolyzed yeast	SmF	CA	Arzumanov et al. (2000) [38]
n-Paraffin	Iron nitrate	SmF	CA	Crolla and Kennedy (2001) ] [44
Ethanol	Ammonium sulfate	SmF	CA	Finogenova et al. (2002) [45]
Glycerol	Yeast extract	SmF	CA	Imandi et al. (2007) [47]
Pineapple residue	Yeast extract	SSF	CA	Imandi et al. (2008) [92]
Sunflower oil	Ammonium sulfate and yeast extract	SmF	CA	Kamzolova et al., (2008) [48]
Sucrose, glucose, and glycerol	Ammonium chloride	SmF	CA	Lazar et al. (2011) [93]
Glycerol	Ammonium sulfate	SmF	CA	Levinson et al. (2007) [46]
Glycerol	Ammonium sulfate and yeast extract	SmF	CA	Papanikolaou et al. (2002) [60]; Makri et al. (2010) [52]
Glucose	Ammonium chloride and yeast extract	SmF	CA	Karasu-Yalcin et al. (2010) [41]
Glucose and stearin	Ammonium sulfate and yeast extract	SmF	CA and lipids	Papanikolaou et al. (2006) [94]
Methanol	Peptone and yeast and malt extracts	SmF	Lipids	Rupčić et al. (1996) [63]
Glucose and olive oil	Peptone and yeast extract	SmF	Lipids and lipase	Najjar et al. (2011) [11]
Industrial fats (stearin)	Peptone and yeast extract	SmF	Lipids	Papanikolaou et al. (2001) [61]
Stearin	Ammonium sulfate and yeast extract	SmF	Lipids, lipase, and biomass	Papanikolaou et al. (2007) [16]
Coconut fat	Ammonium sulfate	SSF	Saturated fatty acid	Parfene et al. (2013) [95]
Sugar cane bagasse	Peptone	SmF	Lipid	Tsigie et al. (2011) [67]
Olive oil, corn oil, and glucose	Urea	SmF	Lipase	Corzo and Revah (1999) [96]
Olive oil	Urea	SmF	CA, lipase, and biomass	Darvishi et al. (2009) [97]
Olive oil	Peptone and yeast extract	SmF	Lipase	Deive et al. (2003) [84]
Olive oil	Peptone	SmF	Lipase	Gonçalves et al. (2013) [98]

CA: citric acid; SmF: submerged fermentation; SSF: solid substrate fermentation.

## References

[1] Demain AL (1999). Pharmaceutically active secondary metabolites of microorganisms. *Applied Microbiology and Biotechnology*.

[2] Torres FAG, Marco JLD, Poças-Fonseca MJ, Felipe MSS, Bon EPS, Ferrara MA, Corvo ML (2008). O uso de leveduras e fungos filamentosos para produção heteróloga de enzimas. *Enzimas em Biotecnologia: Produção, Aplicações e Mercado*.

[3] Spencer JFT, Ragout de Spencer AL, Laluce C (2002). Non-conventional yeasts. *Applied Microbiology and Biotechnology*.

[4] Beckerich J-M, Boisramé-Baudevin A, Gaillardin C (1998). *Yarrowia lipolytica*: a model organism for protein secretion studies. *International Microbiology*.

[5] Kamzolova SV, Shishkanova NV, Morgunov IG, Finogenova TV (2003). Oxygen requirements for growth and citric acid production of *Yarrowia lipolytica*. *FEMS Yeast Research*.

[6] Madzak C, Gaillardin C, Beckerich J-M (2004). Heterologous protein expression and secretion in the non-conventional yeast *Yarrowia lipolytica*: a review. *Journal of Biotechnology*.

[7] Aguedo M, Gomes N, Garcia EE (2005). Decalactone production by *Yarrowia lipolytica* under increased O_2_ transfer rates. *Biotechnology Letters*.

[8] Barth G, Gaillardin C (1997). Physiology and genetics of the dimorphic fungus *Yarrowia lipolytica*. *FEMS Microbiology Reviews*.

[9] Nicaud JM (2012). Yarrowia lipolytica. *Yeast*.

[10] Tai M, Stephanopoulos G (2013). Engineering the push and pull of lipid biosynthesis in oleaginous yeast *Yarrowia lipolytica* for biofuel production. *Metabolic Engineering*.

[11] Najjar A, Robert S, Guérin C, Violet-Asther M, Carrière F (2011). Quantitative study of lipase secretion, extracellular lipolysis, and lipid storage in the yeast *Yarrowia lipolytica* grown in the presence of olive oil: analogies with lipolysis in humans. *Applied Microbiology and Biotechnology*.

[12] Beopoulos A, Cescut J, Haddouche R, Uribelarrea J-L, Molina-Jouve C, Nicaud J-M (2009). *Yarrowia lipolytica* as a model for bio-oil production. *Progress in Lipid Research*.

[13] Coelho MAZ, Amaral PFF, Belo I (2010). lipolytica: an industrial workhorse. *Currient Research, Technology and Education Topics in Applied Microbiology and Microbial Biotechnology*.

[14] Desfougères T, Haddouche R, Fudalej F, Neuvéglise C, Nicaud J-M (2010). SOA genes encode proteins controlling lipase expression in response to triacylglycerol utilization in the yeast *Yarrowia lipolytica*: RESEARCH ARTICLE. *FEMS Yeast Research*.

[15] Fickers P, Benetti P-H, Waché Y (2005). Hydrophobic substrate utilisation by the yeast *Yarrowia lipolytica*, and its potential applications. *FEMS Yeast Research*.

[16] Papanikolaou S, Chevalot I, Galiotou-Panayotou M, Komaitis M, Marc I, Aggelis G (2007). Industrial derivative of tallow: a promising renewable substrate for microbial lipid, single-cell protein and lipase production by *Yarrowia lipolytica*. *Electronic Journal of Biotechnology*.

[17] Lopes M, Gomes N, Mota M, Belo I (2009). *Yarrowia lipolytica* growth under increased air pressure: influence on enzyme production. *Applied Biochemistry and Biotechnology*.

[18] Amaral PFF, Rocha-Leão MHM, Marrucho IM, Coutinho JAP, Coelho MAZ (2006). Improving lipase production using a perfluorocarbon as oxygen carrier. *Journal of Chemical Technology and Biotechnology*.

[19] Suresh S, Srivastava VC, Mishra IM (2009). Techniques for oxygen transfer measurement in bioreactors: a review. *Journal of Chemical Technology and Biotechnology*.

[20] Boopathy R (2000). Factors limiting bioremediation technologies. *Bioresource Technology*.

[21] Vidali M (2001). Bioremediation. An overview. *Pure and Applied Chemistry*.

[22] Ron EZ, Rosenberg E (2002). Biosurfactants and oil bioremediation. *Current Opinion in Biotechnology*.

[23] Scioli C, Vollaro L (1997). The use of *Yarrowia lipolytica* to reduce pollution in olive mill wastewaters. *Water Research*.

[24] Bankar AV, Kumar AR, Zinjarde SS (2009). Environmental and industrial applications of *Yarrowia lipolytica*. *Applied Microbiology and Biotechnology*.

[25] Beopoulos A, Chardot T, Nicaud J-M (2009). *Yarrowia lipolytica*: a model and a tool to understand the mechanisms implicated in lipid accumulation. *Biochimie*.

[26] Christofi N, Ivshina IB (2002). Microbial surfactants and their use in field studies of soil remediation. *Journal of Applied Microbiology*.

[27] Banat IM (1995). Biosurfactants production and possible uses in microbial enhanced oil recovery and oil pollution remiedlation: a review. *Bioresource Technology*.

[28] Cirigliano MC, Carman GM (1984). Isolation of a bioemulsifier from *Candida lipolytica*. *Applied and Environmental Microbiology*.

[29] Sarubbo LA, Marçal MDC, Neves MLC, Silva MDPC, Porto LF, Campos-Takaki GM (2001). Bioemulsifier production in batch culture using glucose as carbon source by *Candida lipolytica*. *Applied Biochemistry and Biotechnology A*.

[30] Braga A, Gomes N, Belo I (2012). Lipase induction in *Yarrowia lipolytica* for castor oil hydrolysis and its effect on *γ*-decalactone production. *Journal of the American Oil Chemists’ Society*.

[31] Aguedo M, Ly MH, Belo I, Teixeira JA, Belin J-M, Waché Y (2004). The use of enzymes and microorganisms for the production of aroma compounds from lipids. *Food Technology and Biotechnology*.

[32] Pagot Y, le Clainche A, Nicaud J-M, Wache Y, Belin J-M (1998). Peroxisomal *β*-oxidation activities and *γ*-decalactone production by the yeast *Yarrowia lipolytica*. *Applied Microbiology and Biotechnology*.

[33] Schrader J, Etschmann MMW, Sell D, Hilmer J-M, Rabenhorst J (2004). Applied biocatalysis for the synthesis of natural flavour compounds—current industrial processes and future prospects. *Biotechnology Letters*.

[34] Anastassiadis S, Aivasidis A, Wandrey C (2002). Citric acid production by *Candida* strains under intracellular nitrogen limitation. *Applied Microbiology and Biotechnology*.

[35] Sauer M, Porro D, Mattanovich D, Branduardi P (2008). Microbial production of organic acids: expanding the markets. *Trends in Biotechnology*.

[36] Papanikolaou S, Aggelis G (2009). Biotechnological valorization of biodiesel derived glycerol waste through production of single cell oil and citric acid by *Yarrowia lipolytica*. *Lipid Technology*.

[37] Rymowicz W, Rywińska A, Zarowska B, Juszczyk P (2006). Citric acid production from raw glycerol by acetate mutants of *Yarrowia lipolytica*. *Chemical Papers*.

[38] Arzumanov TE, Sidorov IA, Shishkanova NV, Finogenova TV (2000). Mathematical modeling of citric acid production by repeated batch culture. *Enzyme and Microbial Technology*.

[39] Papagianni M (2004). Fungal morphology and metabolite production in submerged mycelial processes. *Biotechnology Advances*.

[40] Antonucci S, Bravi M, Bubbico R, di Michele A, Verdone N (2001). Selectivity in citric acid production by *Yarrowia lipolytica*. *Enzyme and Microbial Technology*.

[41] Karasu-Yalcin S, Bozdemir MT, Ozbas ZY (2010). Effects of different fermentation conditions on growth and citric acid production kinetics of two *Yarrowia lipolytica* strains. *Chemical and Biochemical Engineering Quarterly*.

[42] Soccol CR, Vandenberghe LPS, Rodrigues C, Pandey A (2006). New perspectives for citric acid production and application. *Food Technology and Biotechnology*.

[43] Yalcin SK, Bozdemir MT, Ozbas ZY, Mendez-Vilas A (2010). Citric acid production by yeasts: fermentation conditions, process optimization and strain improvement. *Current Research, Technology and Education Topics in Applied Microbiology and Microbial Biotechnology*.

[44] Crolla A, Kennedy KJ (2001). Optimization of citric acid production from *Candida lipolytica* Y-1095 using *n*-paraffin. *Journal of Biotechnology*.

[45] Finogenova TV, Kamzolova SV, Dedyukhina EG (2002). Biosynthesis of citric and isocitric acids from ethanol by mutant *Yarrowia lipolytica* N 1 under continuous cultivation. *Applied Microbiology and Biotechnology*.

[46] Levinson WE, Kurtzman CP, Kuo TM (2007). Characterization of *Yarrowia lipolytica* and related species for citric acid production from glycerol. *Enzyme and Microbial Technology*.

[47] Imandi SB, Bandaru VR, Somalanka SR, Garapati HR (2007). Optimization of medium constituents for the production of citric acid from byproduct glycerol using Doehlert experimental design. *Enzyme and Microbial Technology*.

[48] Kamzolova SV, Finogenova TV, Morgunov IG (2008). Microbiological production of citric and isocitric acids from sunflower oil. *Food Technology and Biotechnology*.

[49] Sarris D, Galiotou-Panayotou M, Koutinas AA, Komaitis M, Papanikolaou S (2011). Citric acid, biomass and cellular lipid production by *Yarrowia lipolytica* strains cultivated on olive mill wastewater-based media. *Journal of Chemical Technology and Biotechnology*.

[50] Dyal SD, Narine SS (2005). Implications for the use of *Mortierella* fungi in the industrial production of essential fatty acids. *Food Research International*.

[51] Rywińska A, Rymowicz W, Zarowska B, Wojtatowicz M (2009). Biosynthesis of citric acid from glycerol by acetate mutants of *Yarrowia lipolytica* in fed-batch fermentation. *Food Technology and Biotechnology*.

[52] Makri A, Fakas S, Aggelis G (2010). Metabolic activities of biotechnological interest in *Yarrowia lipolytica* grown on glycerol in repeated batch cultures. *Bioresource Technology*.

[53] Anastassiadis S, Wandrey C, Rehm H-J (2005). Continuous citric acid fermentation by *Candida oleophila* under nitrogen limitation at constant C/N ratio. *World Journal of Microbiology and Biotechnology*.

[54] Fontanille P, Kumar V, Christophe G, Nouaille R, Larroche C (2012). Bioconversion of volatile fatty acids into lipids by the oleaginous yeast *Yarrowia lipolytica*. *Bioresource Technology*.

[55] Rossi M, Amaretti A, Raimondi S, Leonardi A, Stoytcheva M (2011). Getting lipids for biodiesel production from oleaginous fungi. *Feedstocks and Processing Technologies*.

[56] Wynn JP, Ratledge C (2005). Oils from microorganisms. *Bailey’s Industrial Oil and Fat Products*.

[57] Ratledge C, Cohen Z (2008). Microbial and algal oils: do they have a future for biodiesel or as commodity oils?. *Lipid Technology*.

[58] Ratledge C (2002). Regulation of lipid accumulation in oleaginous micro-organisms. *Biochemical Society Transactions*.

[59] Papanikolaou S, Aggelis G (2011). Lipids of oleaginous yeasts. Part I: biochemistry of single cell oil production. *European Journal of Lipid Science and Technology*.

[60] Papanikolaou S, Chevalot I, Komaitis M, Marc I, Aggelis G (2002). Single cell oil production by *Yarrowia lipolytica* growing on an industrial derivative of animal fat in batch cultures. *Applied Microbiology and Biotechnology*.

[61] Papanikolaou S, Chevalot I, Komaitis M, Aggelis G, Marc I (2001). Kinetic profile of the cellular lipid composition in an oleaginous *Yarrowia lipolytica* capable of producing a cocoa-butter substitute from industrial fats. *Antonie van Leeuwenhoek*.

[62] Ratledge C, Cohen Z (2008). Microbial and algal oils: do they have a future for biodiesel or as commodity oils?. *Lipid Technology*.

[63] Rupčić J, Blagović B, Marić V (1996). Cell lipids of the *Candida lipolytica* yeast grown on methanol. *Journal of Chromatography A*.

[64] Athenstaedt K, Jolivet P, Boulard C (2006). Lipid particle composition of the yeast *Yarrowia lipolytica* depends on the carbon source. *Proteomics*.

[65] Easterling ER, French WT, Hernandez R, Licha M (2009). The effect of glycerol as a sole and secondary substrate on the growth and fatty acid composition of *Rhodotorula glutinis*. *Bioresource Technology*.

[66] Fei Q, Chang HN, Shang L, Choi J-D (2011). Exploring low-cost carbon sources for microbial lipids production by fed-batch cultivation of *Cryptococcus albidus*. *Biotechnology and Bioprocess Engineering*.

[67] Tsigie YA, Wang C-Y, Truong C-T, Ju Y-H (2011). Lipid production from *Yarrowia lipolytica* Po1g grown in sugarcane bagasse hydrolysate. *Bioresource Technology*.

[68] Cescut J (2009). *Accumulation d’acylglycérols par des espèces levuriennes à usage carburant aéronautique: physiologie et performances de procédés*.

[69] Granger LM, Perlot P, Goma G, Pareilleux A (1993). Efficiency of fatty acid synthesis by oleaginous yeasts: prediction of yield and fatty acid cell content from consumed C/N ratio by a simple method. *Biotechnology and Bioengineering*.

[70] Tan KH, Gill CO (1984). Effect of culture conditions on batch growth of Saccharomycopsis lipolytica on olive oil. *Applied Microbiology and Biotechnology*.

[71] Hadeball W (1991). Production of lipase by *Yarrowia lipolytica* I. Lipases from yeasts (Review). *Acta Biotechnologica*.

[72] Glover DJ, McEwen RK, Thomas CR, Young TW (1997). pH-regulated expression of the acid and alkaline extracellular proteases of *Yarrowia lipolytica*. *Microbiology*.

[73] Zarevúcka M, Kejik Z, Saman D, Wimmer Z, Demnevorá K (2005). Enantioselective properties of induced lipases from *Geotrichium*. *Enzyme and Microbial Technology*.

[74] Guerzoni ME, Lanciotti R, Vannini L (2001). Variability of the lipolytic activity in *Yarrowia lipolytica* and its dependence on environmental conditions. *International Journal of Food Microbiology*.

[75] Fickers P, Nicaud JM, Gaillardin C, Destain J, Thonart P (2004). Carbon and nitrogen sources modulate lipase production in the yeast *Yarrowia lipolytica*. *Journal of Applied Microbiology*.

[76] Obradors N, Montesinos JL, Valero F, Lafuente FJ, Sola C (1993). Effects of different fatty acids in lipase production by *Candida rucosa*. *Biotechnology Letters*.

[77] Montesinos JL, Obradors N, Gordillo MA, Valero F, Lafuente J, Solà C (1996). Effect of nitrogen sources in batch and continuous cultures to lipase production by *Candida rugosa*. *Applied Biochemistry and Biotechnology A*.

[78] Domínguez A, Costas M, Longo MA, Sanromán A (2003). A novel application of solid state culture: production of lipases by *Yarrowia lipolytica*. *Biotechnology Letters*.

[79] Dalmau E, Montesinos JL, Lotti M, Casas C (2000). Effect of different carbon sources on lipase production by *Candida rugosa*. *Enzyme and Microbial Technology*.

[80] Sharma R, Chisti Y, Banerjee UC (2001). Production, purification, characterization, and applications of lipases. *Biotechnology Advances*.

[81] Roveda M, Hemkemeier M, Crolla LM (2010). Avaliação da produção de lipases por diferentes cepas de microrganismos isolados em efluentes de laticínios por fermentação submersa. *Ciência e Tecnologia de Alimentos*.

[82] Tan T, Zhang M, Wang B, Ying C, Deng L (2003). Screening of high lipase producing *Candida* sp. and production of lipase by fermentation. *Process Biochemistry*.

[83] Shirazi SH, Rahman SR, Rahman MM (1998). Short communication: production of extracellular lipases by *Saccharomyces cerevisiae*. *World Journal of Microbiology and Biotechnology*.

[84] Deive FJ, Costas M, Longo MA (2003). Production of a thermostable extracellular lipase by *Kluyveromyces marxianus*. *Biotechnology Letters*.

[85] Freitas L, Bueno T, Perez VH, Santos JC, de Castro HF (2007). Enzymatic hydrolysis of soybean oil using lipase from different sources to yield concentrated of polyunsaturated fatty acids. *World Journal of Microbiology and Biotechnology*.

[86] Lotti M, Monticelli S, Luis Montesinos J, Brocca S, Valero F, Lafuente J (1998). Physiological control on the expression and secretion of *Candida rugosa* lipase. *Chemistry and Physics of Lipids*.

[87] Gurvitz A, Rottensteiner H (2006). The biochemistry of oleate induction: transcriptional upregulation and peroxisome proliferation. *Biochimica et Biophysica Acta*.

[88] Destain J, Roblain D, Thonart P (1997). Improvement of lipase production from *Yarrowia lipolytica*. *Biotechnology Letters*.

[89] Treichel H, de Oliveira D, Mazutti MA, di Luccio M, Oliveira JV (2010). A review on microbial lipases production. *Food and Bioprocess Technology*.

[90] Salihu A, Alam MZ, AbdulKarim MI, Salleh HM (2012). Lipase production: an insight in the utilization of renewable agricultural residues. *Resources, Conservation and Recycling*.

[91] Almeida AF, Taulk-Tomisielo SM, Carmona EC (2012). Influence of carbon and nitrogen sources on lipase production by a newly isolated *Candida viswanathii* strain. *Annals of Microbiology*.

[92] Imandi SB, Bandaru VVR, Somalanka SR, Bandaru SR, Garapati HR (2008). Application of statistical experimental designs for the optimization of medium constituents for the production of citric acid from pineapple waste. *Bioresource Technology*.

[93] Lazar Z, Walczak E, Robak M (2011). Simultaneous production of citric acid and invertase by *Yarrowia lipolytica* SUC^+^ transformants. *Bioresource Technology*.

[94] Papanikolaou S, Galiotou-Panayotou M, Chevalot I, Komaitis M, Marc I, Aggelis G (2006). Influence of glucose and saturated free-fatty acid mixtures on citric acid and lipid production by *Yarrowia lipolytica*. *Current Microbiology*.

[95] Parfene G, Horincar V, Tyagi AK, Malik A, Bahrim G (2013). Production of medium chain saturated fatty acids with enhanced antimicrobial activity from crude coconut fat by solid state cultivation of *Yarrowia lipolytica*. *Food Chemistry*.

[96] Corzo G, Revah S (1999). Production and characteristics of the lipase from *Yarrowia lipolytica* 681. *Bioresource Technology*.

[97] Darvishi F, Nahvi I, Zarkesh-Esfahani H, Momenbeik F (2009). Effect of plant oils upon lipase and citric acid production in *Yarrowia lipolytica* yeast. *Journal of Biomedicine and Biotechnology*.

[98] Gonçalves FAG, Colen G, Takahashi JA (2013). Optimization of cultivation conditions for extracellular lipase production by *Yarrowia lipolytica* using response surface method. *African Journal of Biotechnology*.

[99] Hiol A, Jonzo MD, Rugani N, Druet D, Sarda L, Comeau LC (2000). Purification and characterization of an extracellular lipase from a thermophilic *Rhizopus oryzae* strain isolated from palm fruit. *Enzyme and Microbial Technology*.

[100] Carlile M, Watkinson SC (1997). *The Fungi*.

[101] Colen G (2006). *Isolamento e seleção de fungos filamentosos produtores de lipases [Ph.D. thesis]*.

[102] Jennings DH (1995). *The Physiology of Fungal Nutrition*.

[103] Alves MH, Campos-Takaki GM, Figueiredo Porto AL, Milanez AI (2002). Screening of *Mucor spp.* for the production of amylase, lipase, polygalacturonase and protease. *Brazilian Journal of Microbiology*.

[104] Bussamara R, Fuentefria AM, Oliveira ESD (2010). Isolation of a lipase-secreting yeast for enzyme production in a pilot-plant scale batch fermentation. *Bioresource Technology*.

[105] Sathish Yadav KN, Adsul MG, Bastawde KB, Jadhav DD, Thulasiram HV, Gokhale DV (2011). Differential induction, purification and characterization of cold active lipase from *Yarrowia lipolytica* NCIM 3639. *Bioresource Technology*.

